# SRLST: a unified multimodal representation learning framework for spatial transcriptomics analysis

**DOI:** 10.1093/bioinformatics/btag524

**Published:** 2026-07-21

**Authors:** Wei Lan, Xiao Deng, Tongsheng Ling, Guohang He, Xuhua Yan, Ruiqing Zheng, Min Li, Shirui Pan, Yi Pan

**Affiliations:** Guangxi Key Laboratory of Multimedia Communications and Network Technology, School of Computer, Electronic and Information, Guangxi University, Nanning, Guangxi, China; Guangxi Key Laboratory of Multimedia Communications and Network Technology, School of Computer, Electronic and Information, Guangxi University, Nanning, Guangxi, China; Guangxi Key Laboratory of Multimedia Communications and Network Technology, School of Computer, Electronic and Information, Guangxi University, Nanning, Guangxi, China; Guangxi Key Laboratory of Multimedia Communications and Network Technology, School of Computer, Electronic and Information, Guangxi University, Nanning, Guangxi, China; Guangxi Key Laboratory of Multimedia Communications and Network Technology, School of Computer, Electronic and Information, Guangxi University, Nanning, Guangxi, China; School of Computer and Engineering, Central South University, Changsha, Hunan, China; School of Computer and Engineering, Central South University, Changsha, Hunan, China; School of Information and Communication Technology, Griffith University, Southport, Queensland, Australia; School of Computer Science and Control Engineering, Shenzhen University of Advanced Technology, Shenzhen, Guangdong, China

## Abstract

**Motivation:**

Spatial transcriptomics (ST) enables molecular profiling within native tissue architecture, yet accurate delineation of spatial domains in ST data is challenging, as it demands the coordinated integration of transcriptomic, spatial, and tissue histological information.

**Results:**

We present SRLST, an unsupervised representation learning framework that holistically harmonize these three complementary data modalities to precisely uncover tissue organization. SRLST employs a dual-graph variational autoencoding strategy to jointly model spatial proximity and morphological relations, fusing these with gene-expression embeddings into a unified latent space. Across distinct experimental datasets, SRLST consistently outperforms existing methods in delineating cortical organization, identifying small discontinuous tissue compartments, and capturing complex intratumor heterogeneity.

**Availability and implementation:**

The code implementation of the SRLST algorithm is available at https://github.com/lanbiolab/SRLST.

## 1 Introduction

Spatial transcriptomics (ST) has revolutionized the way we study gene expression within its native tissue context by simultaneously measuring transcript abundance and preserving the spatial organization of cells (Asp *et al.* 2020, Rao *et al.* 2021, Palla *et al.* 2022). This emerging technology enables researchers to explore not only the quantitative profiles of gene expression but also the intricate spatial relationships among cells and the dynamic architecture of tissue microenvironments (Longo *et al.* 2021). Such integrative information has provided unprecedented insights into fundamental biological processes, including tissue development, cell differentiation, and disease progression (Lein *et al.* 2017, Yoosuf *et al.* 2020, Maynard *et al.* 2021).

Current ST platforms can be broadly categorized into image-based and next-generation sequencing (NGS)-based approaches. Image-based techniques such as MERFISH (Lu *et al.* 2021), seqFISH (Lubeck *et al.* 2014), and STARmap (Wang *et al.* 2018) utilize high-resolution imaging to directly localize specific RNA molecules through fluorescence in situ hybridization, achieving single-cell or even subcellular precision but often limited to a restricted set of genes. In contrast, NGS-based methods, including 10× Visium, Slide-seq (Rodriques *et al.* 2019), and Stereo-seq (Chen *et al.* 2022), leverage spatial barcoding to capture a transcriptome-wide view, yet their spatial resolution is constrained by the spot size on the capture array (Xu *et al.* 2023). These complementary trade-offs highlight a persistent gap between molecular coverage and spatial precision, motivating the development of advanced computational frameworks that can effectively integrate multimodal information embedded in ST data (Zhao *et al.* 2024).

A central goal of ST data analysis is to delineate spatial domains that correspond to distinct histological or functional tissue compartments. Numerous computational approaches have been proposed to address this task (Berglund *et al.* 2018, Thrane *et al.* 2018). Representative methods include SpaGCN (Hu *et al.* 2021), which integrates gene expression, spatial location, and histology image features using graph convolutional networks; STAGATE (Dong and Zhang 2022), which employs graph attention autoencoders to adaptively model spatial similarity; DeepST (Xu *et al.* 2022), which combines graph neural networks with denoising autoencoders to enhance domain segmentation; stLearn (Pham *et al.* 2023), which reconstructs spatial trajectories via pseudo-time inference; and SEDR (Xu *et al.* 2024), which jointly embeds gene expression and spatial topology through variational graph autoencoders. PROST (Liang *et al.* 2024) utilizes self-attention mechanisms for spatial domain identification. GraphST (Long *et al.* 2023) employs graph contrastive learning to integrate spatial transcriptomics and histology features for enhanced domain segmentation. More recently, SpaMask (Min *et al.* 2025) employs a dual masking graph autoencoder with contrastive learning to enhance robustness against noise, SpaICL (Zhao and Min 2025) adopts an image-guided curriculum learning strategy to integrate multimodal features, mclSTExp (Min *et al.* 2024) leverages multimodal contrastive learning for spatial gene expression prediction from histology images, SpaCross (Fang and Min 2025) introduces a cross-masked graph autoencoder with adaptive spatial-semantic fusion for multi-slice integration, SpaBatch (Niu *et al.* 2025) focuses on 3D spatial domain identification and batch effect correction across multiple slices and SpaRank (Yan *et al.* 2026) introduced a transferable approach for spatial omics deconvolution. In addition, unsupervised representation learning methods such as Lan *et al.* (2026a) proposed an adversarial autoencoder-based framework for spatial transcriptomics analysis, and scMoMtF (Lan *et al.* 2024a) provides an interpretable multi-task framework for single-cell multi-omics integration, achieving heterogeneous data integration. Beyond individual methods, recent surveys have provided comprehensive overviews of transformer-based architectures in single-cell data modeling (Lan *et al.* 2024b) and large language models for biomedical data analysis (Lan *et al.* 2025a), offering valuable resources for understanding the broader methodological landscape in this rapidly evolving field. In general, spatial coordinates combined with gene expression profiles indeed help recover tissue compartments. However, spatial proximity does not always correspond to biological similarity, particularly in tissues with sharp boundaries or heterogeneous architectures. Histological images therefore provide essential morphological information missed by spatial-expression models. However, existing histology-informed methods largely simplify image features into spatial constraints rather than a biologically informative modality, leading to over-smoothed boundaries and failure to preserve small or discontinuous domains.

To address these limitations, we introduce SRLST, an unsupervised spatial representation learning framework that jointly model gene expression, spatial coordinates, and histological morphology features. To jointly encode spatial proximity and morphological similarity between spots, SRLST constructs two complementary K-nearest neighbor graphs, one based on spatial coordinates and the other on histological image features, and applies variational graph autoencoders to learn biologically meaningful latent representations that preserve both spatial and morphological information. Through extensive evaluation across human brain, liver, breast cancer, and zebrafish melanoma datasets, SRLST consistently improves spatial domain identification and downstream biological interpretations, offering a reliable computational framework for uncovering tissue organization and microenvironmental heterogeneity.

## 2 Methods

### 2.1 Input of SRLST

SRLST takes three inputs: spatial coordinates, gene expression matrices, and corresponding histological images.

### 2.2 Histological image aggregation

To extract tissue morphology features from histological images, we aggregate local pixel information around each spatial spot. For spot i with center coordinate $\left(x_{i},y_{i}\right)$, the mean RGB intensities within a 30×30 window centered at the pixel are calculated to obtain its image-derived feature vector $\left[r_{i},g_{i},b_{i}\right]$. Repeating this process for all N spots yields the aggregated morphology feature matrix T:


(1) $$T=[\begin{array}{ccc} r_{1} & g_{1} & b_{1}\\ r_{2} & g_{2} & b_{2}\\ \vdots & \vdots & \vdots \\ r_{N} & g_{N} & b_{N} \end{array}],T\in R^{N\times 3}$$

### 2.3 Graph construction

To characterize spatial proximity and morphology similarity between spots, we compute pairwise Euclidean distances using the spatial coordinate matrix $S\in R^{N\times 2}$ and the morphology feature matrix $T\in R^{N\times 3}$. For each spot, the K nearest neighbors (K=12 by default) are selected to construct an undirected spatial adjacency graph $A^{S}$ and a morphology adjacency graph $A^{T}$, respectively. Specifically, if i and j are neighbors, $A_{ij}$ = $A_{ji}$ = 1, otherwise 0.

### 2.4 Gene expression matrix processing

For gene expression matrix $X\in R^{N\times G}$ (G is the number of genes), we use SCANPY to filter low-expression genes (the number of expressing cells ≤ 50 and the total count ≤ 10 which are used for all datasets) and eliminate the differences in sequencing depth through library size normalization. Subsequently, we select 2000 highly variable genes, apply *Z*-score scaling to normalized counts, and perform PCA to obtain 200 principal components. The resulting matrix $X^{\prime }\in R^{N\times 200}$ is used as input for downstream analyses.

### 2.5 Representation learning

SRLST applies a multi-layer neural network, E, to transform the preprocessed matrix $X^{\prime }$ into a low-dimensional feature embedding $Z_{f}$:


(2) $$Z_{f}=E(X^{\prime })\in R^{N\times D_{f}}$$

Two variational graph autoencoders were used to encode spatial and morphology relations, denoted as $G_{S}$ and $G_{T}$, respectively. Both are implemented using MFConv-based graph neural networks. $G_{S}$ takes the spatial adjacency graph $A^{S}$ and the feature embedding $Z_{f}$ as inputs to learn embedding $Z_{s}$, while $G_{T}$ takes the morphology adjacency graph $A^{T}$ and $Z_{f}$ to learn embedding $Z_{T}$. The encoder of each VGAE consists of two layers. For the encoder $G_{S}$, the first layer is defined as:


(3) X¯S=GS1(Zf,AS)=ReLU(A∼SZfW1S)

(4) A∼S=DS-12A^SDS-12

Where ${\tilde{A}}^{S}$ is the symmetrically normalized adjacency matrix; ${\hat{A}}^{S}=A^{S}+I$; $D_{s}$ represents the degree matrix of ${\hat{A}}^{S}$. The second layer outputs the mean $\mu_{s}$ and log-variance $\log\sigma_{s}^{2}$:


(5) $$\mu_{S}=G_{S∼\mu }^{2}(Z_{f},A^{S})={\tilde{A}}^{S}{\overset{‾}{X}}_{S}W_{\mu }^{S}$$

(6) $$\log\sigma_{S}^{2}=G_{S∼\sigma }^{2}(Z_{f},A^{S})={\tilde{A}}^{S}{\overset{‾}{X}}_{S}W_{\sigma }^{S}$$

Where $W_{\mu }^{S}$ and $W_{\sigma }^{S}$ are learnable weight parameters. The latent embedding $Z_{s}$ is then sampled as:


(7) $$Z_{s}=\mu_{S}+\sigma_{S}^{2}*\varepsilon ,Z_{s}\in R^{N\times D_{s}}$$

Where hyperparameter $D_{s}$ is the dimension of $Z_{s}$ and ε∼N(0,1).

Similar to the construction of $Z_{s}$, the construction of $Z_{t}$ is as follows:


(8) $${\overset{‾}{X}}_{T}=G_{T}^{1}(Z_{f},A^{T})=\mathrm{ReLU}({\tilde{A}}^{T}Z_{f}W_{1}^{T})$$

(9) $${\tilde{A}}^{T}=D_{T}^{-\frac{1}{2}}{\hat{A}}^{T}D_{T}^{-\frac{1}{2}}$$

(10) $$\mu_{T}=G_{T∼\mu }^{2}(Z_{f},A^{T})={\tilde{A}}^{T}{\overset{‾}{X}}_{T}W_{\mu }^{T}$$

(11) $$\log\sigma_{T}^{2}=G_{T∼\sigma }^{2}(Z_{f},A^{T})={\tilde{A}}^{T}{\overset{‾}{X}}_{T}W_{\sigma }^{T}$$

(12) $$Z_{t}=\mu_{T}+\sigma_{T}^{2}*\varepsilon ,Z_{t}\in R^{N\times D_{t}}$$

The final latent representation $Z\in R^{N\times (D_{f}+D_{s}+D_{t})}$ is obtained by concatenating the feature embedding $Z_{f}$, spatial relation embedding $Z_{s}$, and morphology embedding $Z_{t}$ along the feature dimension.

### 2.6 Loss function

The loss function of SRLST consists of three components: (i) graph reconstruction loss for spatial and morphology graphs, (ii) expression reconstruction loss, and (iii) the deep embedded clustering (DEC) loss. The spatial graph reconstruction loss is defined as:


(13) $$L_{s}=E_{q(Z_{s}|A^{S},Z_{f})}[\log\;p(A^{S}|Z_{s})]-\mathrm{KL}[q(Z_{s}|A^{S},Z_{f})∥p(Z_{s})]$$

(14) $$p(A^{S}|Z_{s})=\prod_{i,j}\sigma (z_{s,i}^{T}z_{s,j})$$

(15) $$p(Z_{s})=\prod_{i=1}^{N}N(z_{s,i}|0,I)$$

Where σ represents sigmoid function. The morphology reconstruction loss $L_{t}$ is defined analogously by replacing the spatial graph $A^{S}$ with the morphology graph $A^{T}$ and the corresponding latent variables $Z_{s}$ with $Z_{t}$.

The reconstruction loss of gene expression is defined as:


(16) $$L_{g}=\frac{1}{N}\sum_{i=1}^{N}∥{\hat{x}}_{i}-x_{i}|{|}^{2}$$

Where ${\hat{x}}_{i}$ is obtained by applying a decoder D(⋅) on the final latent embedding $z_{i}$.

For DEC loss, we first use the KMeans algorithm implemented in scikit-learn to initialize the cluster centers. The number of clusters is predefined as a hyperparameter K. The calculation of DEC loss consists of two steps. First, the soft assignment probability $q_{ij}$ of latent embedding $z_{i}\in Z$ (i = 1, 2, …, N) assigned to the cluster center $\mu_{j}$ (j = 1, 2, …, K) is defined as:


(17) $$q_{ij}=\frac{{\left(1+\frac{{∥z_{i}-\mu_{j}∥}^{2}}{\alpha }\right)}^{-1}}{\sum_{j^{\prime }=1}^{K}{\left(1+\frac{{∥z_{i}-\mu_{j^{\prime }}∥}^{2}}{\alpha }\right)}^{-1}}$$

Where α is a predefined constant. Then, we calculate the auxiliary target distribution $p_{ij}$ based on $q_{ij}$. The auxiliary target distribution $p_{ij}$ guides the model to focus on highly reliable samples and optimize the cluster structure through confidence enhancement and balanced normalization:


(18) $$p_{ij}=\frac{q_{ij}^{2}/\sum_{i=1}^{n}q_{ij}}{\sum_{j^{\prime }=1}^{K}\left(q_{ij^{\prime }}^{2}/\sum_{i=1}^{n}q_{ij^{\prime }}\right)}$$

The DEC loss requires predictions towards high-confidence targets by minimizing the KL divergence of $q_{ij}$ and $p_{ij}$:


(19) $$L_{\mathrm{dec}}=\mathrm{KL}(p∥q)=\sum_{i=1}^{N}\sum_{j=1}^{K}p_{ij}l\mathrm{og}\frac{p_{ij}}{q_{ij}}$$

Finally, all model components, including the pretrained VGAE encoders and the expression autoencoder, are jointly fine-tuned by minimizing the combined loss function. The combined loss function is defined as:


(20) $$L_{\mathrm{final}}=L_{s}+L_{t}+L_{g}+L_{\mathrm{dec}}$$

## 3 Results

### 3.1 Overall of SRLST

**Figure 1:**
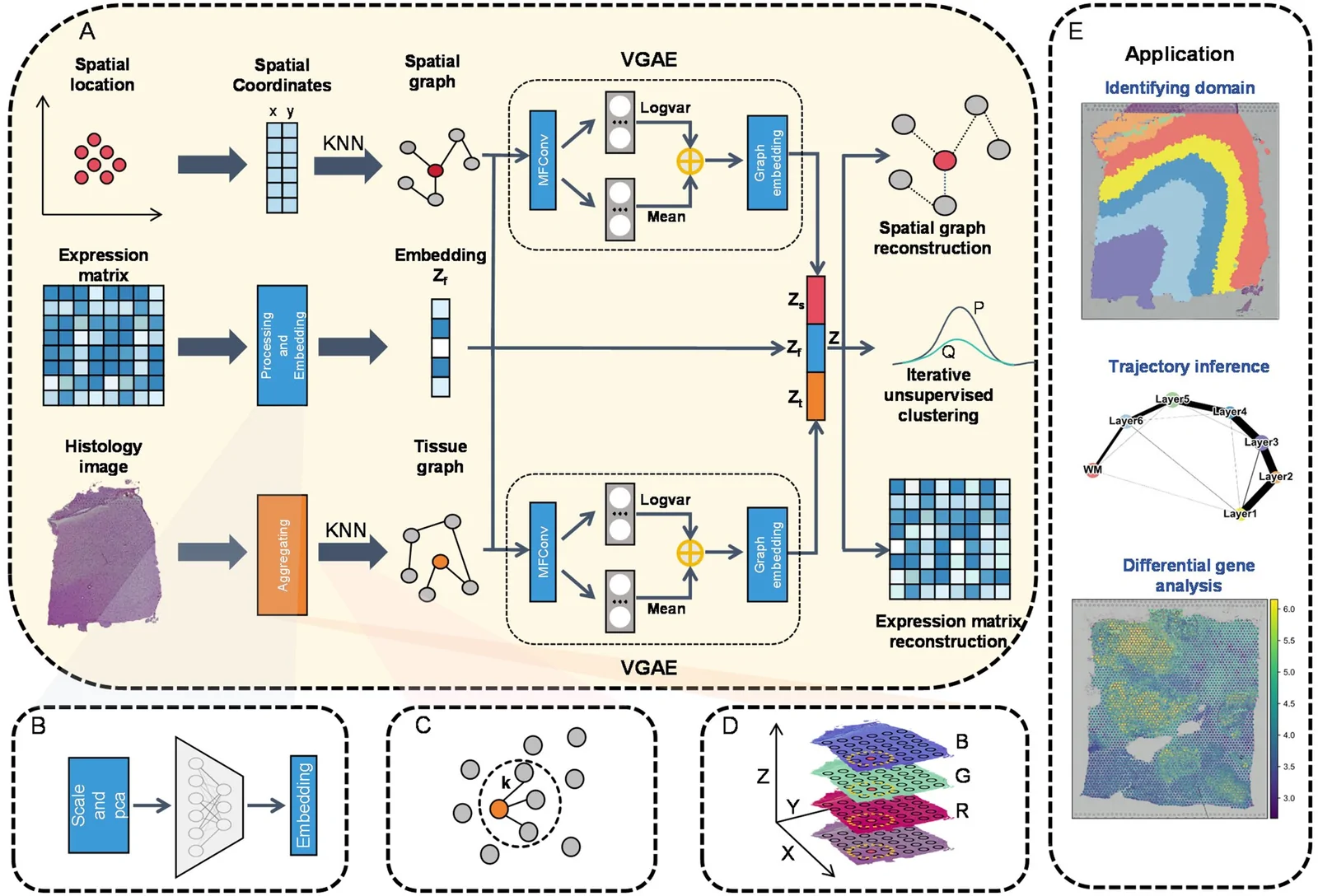
Overview of SRLST. (A) Unsupervised workflow integrating spatial coordinates, gene expression, and histology to learn a unified latent space for ST data. (B) Gene expression is preprocessed and embedded by a multi-layer network. (C) Spatial and morphological neighbor relationships are established using *k*-NN based on spot coordinates and histology-derived features, respectively. (D) Local tissue morphology is represented as multi-channel texture features derived from RGB histology images. (E) The latent representation supports various downstream analyses, including spatial domain calling, visualization, trajectory inference, and differential expression.

SRLST leverages a dual-graph variational autoencoding strategy that models spatial proximity and tissue morphology in parallel, enabling a unified latent representation that integrates gene expression, spatial relationships, and histological structure (Fig. 1). To jointly capture spatial proximity and morphological similarity, the model constructs two complementary *K*-nearest neighbor (KNN) graphs: a spatial adjacency graph, defined by the physical coordinates of the spots, and a morphological adjacency graph, defined by histological features computed as the mean RGB intensities within local pixel neighborhoods. The spatial and morphology adjacency graphs are independently captured by parallel VGAEs to extract their distinct structural information. These spatial and morphological embeddings are then integrated with the embeddings of expression data, co-mapping the entire feature set into a unified latent space.

SRLST is trained in two sequential stages. In the pretraining stage, the model jointly reconstructs the spatial and morphology graphs to capture intrinsic structural patterns, providing a self-supervised initialization for the subsequent clustering task. In the clustering stage, a deep embedded clustering (DEC) objective is introduced to refine the learned unified representations toward compact and biologically meaningful clusters. Through this two-stage optimization, SRLST effectively delineates fine-grained tissue boundaries while preserving global spatial continuity, providing a robust foundation for downstream analyses such as spatial domain identification, visualization, and differential expression analysis.

### 3.2 SRLST accurately delineates cortical laminar architecture in human DLPFC spatial transcriptomics data

**Figure 2:**
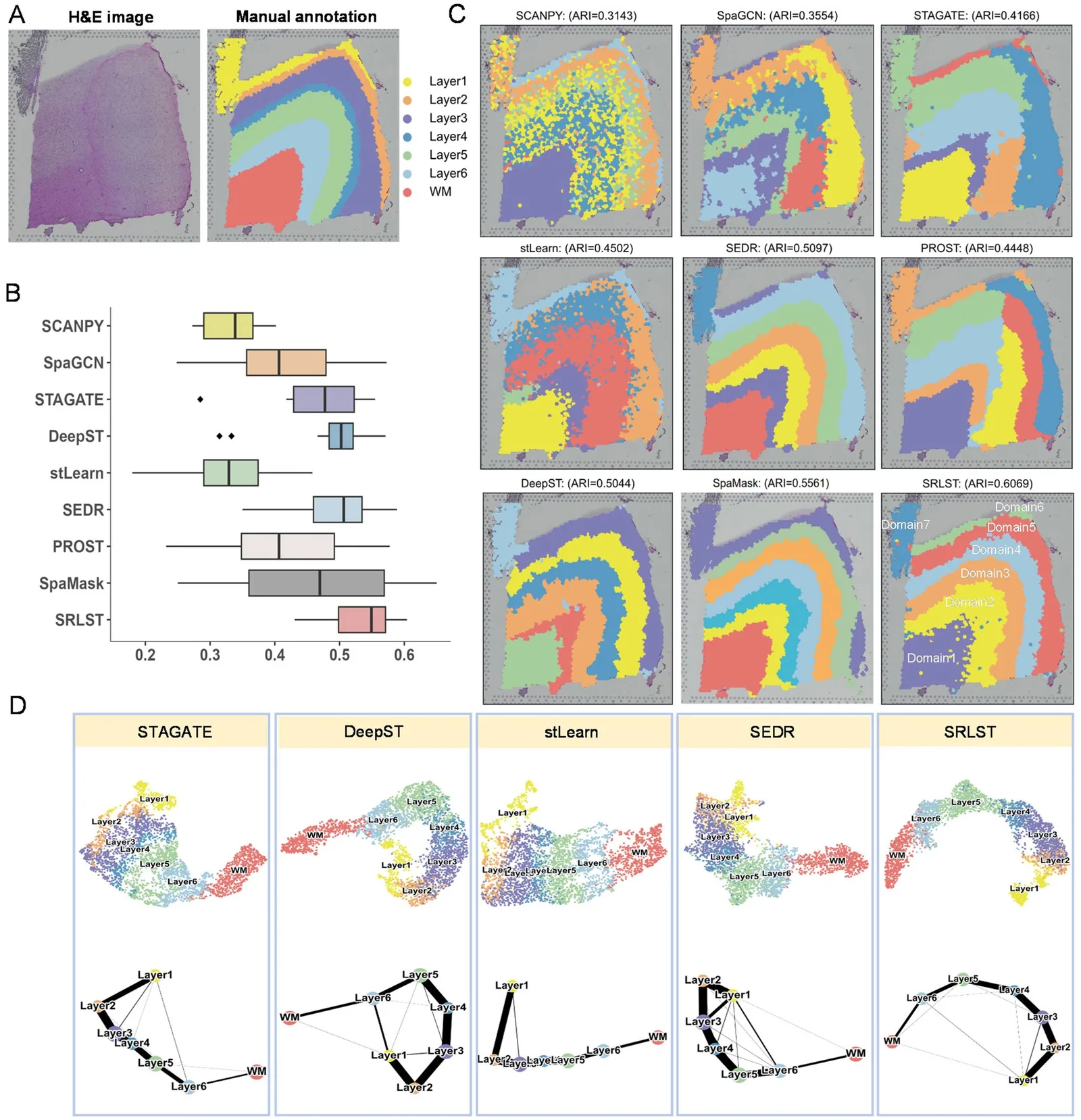
Benchmarking SRLST against existing spatial methods on the DLPFC dataset. (A) H&E image and expert annotations for slice 151675. (B) Comparison of ARIs scores of all methods across 12 DLPFC slices. Lower and upper hinges, first and third quartiles (Q1, Q3); whiskers, range of 1.5-times the interquartile; Centre line, median; Dot, outliers. (C) Spatial clustering results of all methods on slice 151675. (D) UMAP embeddings and PAGA graph built based on embeddings of STAGATE, DeepST, stLearn, SEDR and SRLST on slice 151675.

We first assessed the performance of SRLST to identify spatial domains using the human dorsolateral prefrontal cortex (DLPFC) dataset from the 10× Genomics Visium platform. This dataset contains twelve cortical sections manually annotated by Maynard *et al.* (2021) into six cortical layers (L1–L6) and white matter (WM) (with section 151675 shown as an example in Fig. 2A). Using these annotations as ground truth, we compared our method against the expression-based tool Scanpy (Wolf *et al.* 2018) and several spatially aware models, including SpaGCN (Hu *et al.* 2021), STAGATE (Dong and Zhang 2022), DeepST (Xu *et al.* 2022), stLearn (Pham *et al.* 2023), SEDR (Xu *et al.* 2024), PROST (Liang *et al.* 2024) and SpaMask (Min *et al.* 2025). We quantified the agreement between predicted and annotated domains using the adjusted rand index (ARI), which ranges from 0 (random agreement) to 1 (perfect concordance).

Across the twelve sections, SRLST consistently achieved the highest overall ARI score (Yuan *et al.* 2024) (mean = 0.53), outperforming all compared methods (Fig. 2B). The expression-based tool Scanpy produced the lowest ARI scores among all benchmarks, markedly below those of methods incorporating spatial information, indicating that spatial information is critical for accurate delineation of tissue domains. To further illustrate this improvement, we examined section 151675 of the DLPFC dataset as a representative example. To assess the stability of our method, we performed 10 independent runs with different random seeds on section 151675. SRLST achieved a mean ARI of 0.607 ± 0.015 (mean ± standard deviation) and accurately delineated six cortical layers (L1–L6) and the white matter whose boundaries aligned closely with the histological annotations (Fig. 2C). In contrast, Scanpy, which relies solely on gene expression, failed to recover the laminar architecture, whereas SpaGCN and stLearn produced discontinuous boundaries with scattered misclassified spots. STAGATE and PROST generated sharper partitions but deviated substantially from manual segmentation, while DeepST and SEDR captured the overall laminar organization but failed to clearly distinguish adjacent cortical layers. SpaMask, despite its robustness-enhancing design, tended to partition the same layer into discontinuous and fragmented regions, failing to preserve spatial continuity.

To further investigate the learned representations, we examined the low-dimensional embeddings generated from different methods by uniform manifold approximation and projection (UMAP) (McInnes *et al.* 2018). UMAP visualization of SRLST’s embeddings revealed well-segregated clusters that corresponded precisely to the annotated cortical layers (Fig. 2D). In contrast, UMAP of STAGATE, stLearn, and SEDR showed extensive mixing among upper layers (L1–L3), indicating that these models failed to preserve fine-grained laminar boundaries. Trajectory inference using partition-based graph abstraction (PAGA) (Wolf *et al.* 2019) analysis showed that SRLST embeddings recovered a smooth and nearly linear transition from layer 1 to white matter, consistent with the expected laminar organization of the cortex. In contrast, trajectories derived from DeepST and SEDR exhibited weak continuity between layers and failed to capture the hierarchical progression across cortical depths. Together, these results demonstrate that SRLST learns spatial representations that preserve hierarchical tissue organization and delineate biologically meaningful boundaries with high accuracy.

### 3.3 SRLST improves detection of small and discontinuous tissue compartments in liver tissue

**Figure 3:**
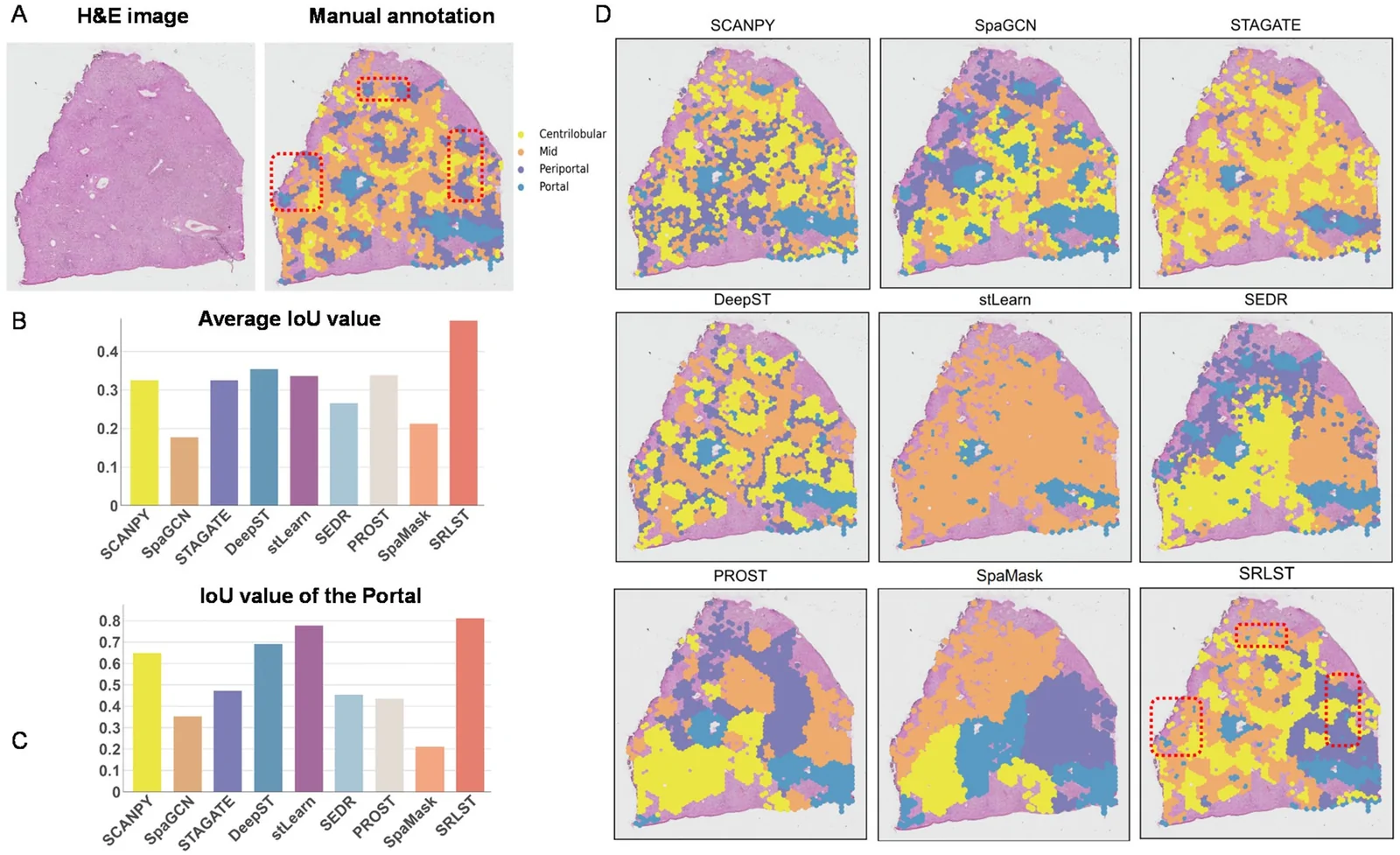
Identification of small and spatially fragmented portal regions in liver tissue. (A) H&E image and expert annotations, with small portal regions highlighted within red boxes. (B) Comparison of average IoU scores of all methods. (C) IoU scores of all methods on portal regions. (D) Spatial clustering results of all compared methods. Portal-associated clusters identified by SRLST are highlighted.

To evaluate whether SRLST can accurately resolve spatial domains in tissues with fragmented and discontinuous compartments, we analyzed a human liver spatial transcriptomics dataset generated using the 10× Genomics Visium platform. The dataset includes four histologically defined regions (centrilobular, midzonal, periportal, and portal) that were annotated in the original study based on hematoxylin and eosin (H&E) staining (Fig. 3A). Unlike the relatively continuous layers of the cerebral cortex (Yuan *et al.* 2024), liver lobules exhibit discontinuous and irregular structures, particularly in the small portal areas that are sparsely distributed throughout the tissue (Guilliams *et al.* 2022). Such non-continuous regions are challenging for graph-based spatial methods, which often rely on smooth neighborhood relationships.

To evaluate segmentation accuracy, we used the Intersection over Union (IoU) metric (Chen *et al.* 2023). IoU is defined as the area of overlap between predicted domains ($D_{\mathrm{pre}}$) and annotated domains ($D_{\mathrm{true}}$) divided by the area of their union ($\left|D_{\mathrm{pre}}\cap D_{\mathrm{true}}|÷|D_{\mathrm{pre}}\cup D_{\mathrm{true}}\right|$), with higher values indicating better spatial segmentation accuracy. IoU penalizes both over-smoothing and fragmentation, making it suitable for tissues with fragmented regions. For each histological region, IoU was calculated independently and then averaged across domains to obtain the overall score. We compared SRLST with SCANPY, SpaGCN, STAGATE, DeepST, stLearn, SEDR, and PROST.

Across all regions, SRLST achieved the highest segmentation accuracy, with a mean IoU of 0.47 (Fig. 3B). In the portal region, which contains small and discontinuous structures, SRLST reached an IoU of 0.82, surpassing stLearn (0.78) and DeepST (0.70). Although these methods performed reasonably well on portal areas, their mean IoUs across all regions were below 0.40 (Fig. 3C). SCANPY, SpaGCN, STAGATE, SEDR, PROST and SpaMask showed even lower scores, often failing to delineate small boundaries. Interestingly, SCANPY, despite lacking spatial modeling, performed slightly better than SpaGCN and SEDR in discontinuous regions, suggesting that excessive spatial smoothing in some graph-based methods can obscure fine structural details.

Visual inspection of spatial domain maps showed that SRLST closely reproduced the histological annotations (Fig. 3D). The model accurately delineated the portal regions, including the small and spatially separated clusters highlighted by red boxes in Fig. 3A. In contrast, most compared methods produced oversmoothed segmentations that blended small portal domains with neighboring areas. SpaMask failed to capture discontinuous regions altogether, stLearn, DeepST, SpaGCN, and STAGATE captured the main portal zones but missed smaller fragments, whereas SEDR and PROST yielded overly uniform maps that obscured structural boundaries.

Together, these results demonstrate that SRLST effectively preserves spatial discontinuities and accurately delineates small tissue structures, underscoring its applicability to complex and heterogeneous tissues such as the liver.

### 3.4 SRLST enhances delineation of intratumor heterogeneity in breast cancer

**Figure 4:**
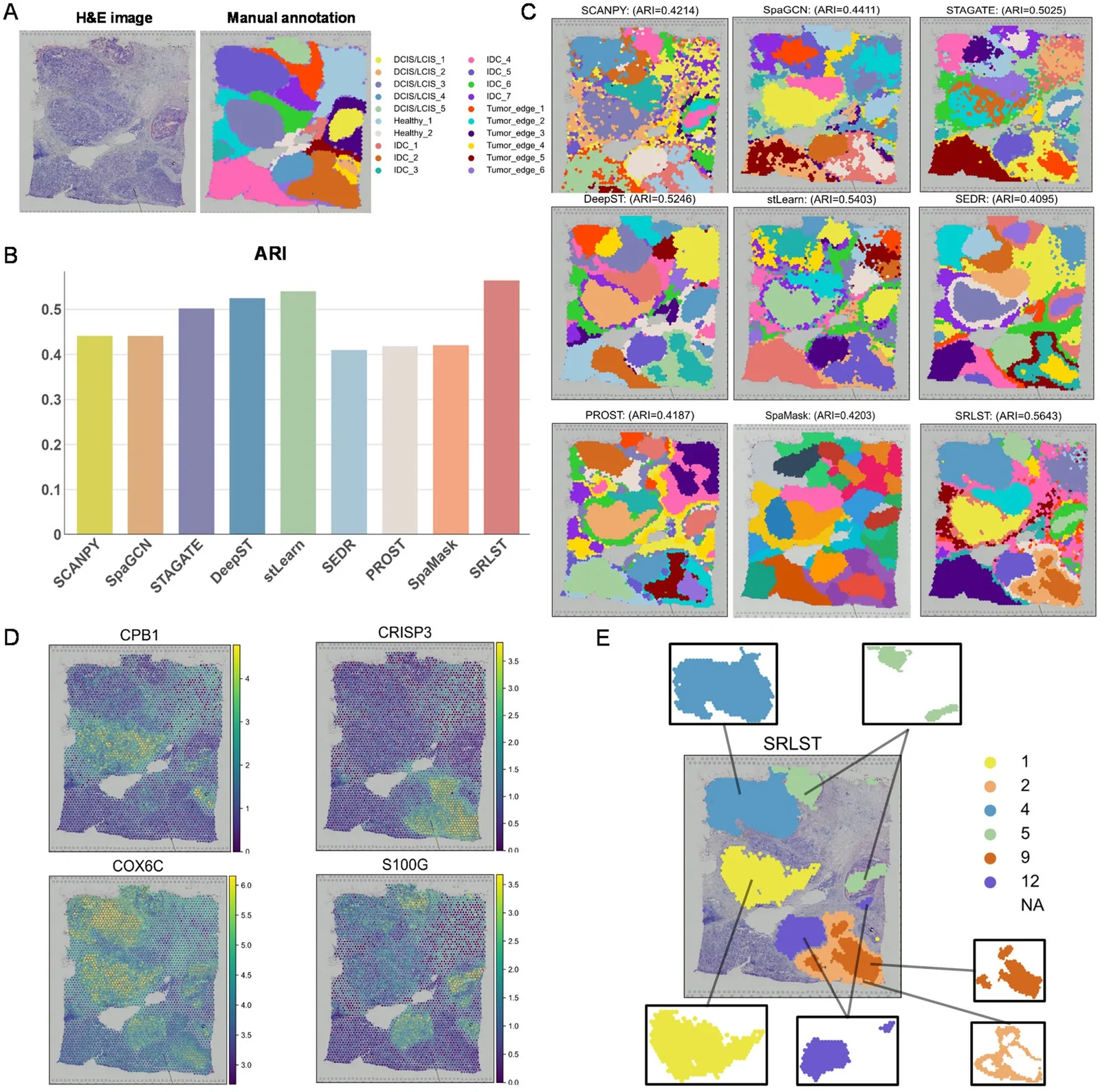
Spatial domain delineation and marker localization in breast cancer tissue. (A) H&E image and expert annotations. (B) ARI comparison among all compared methods. (C) Spatial clustering results of all methods. (D) Spatial expression of genes CPB1, CRISP3, COX6X, and S100G. (E) Visualization of selected tumor-related domains (1, 2, 4, 5, 9, and 12).

Breast cancer remains the most prevalent malignancy worldwide (Fuentes *et al.* 2024), and characterizing its intra-tumor heterogeneity is crucial for understanding disease progression and guiding therapy (Wang *et al.* 2024). To evaluate the capability of SRLST in complex tumor microenvironments, we analyzed a human breast cancer dataset generated using the 10× Genomics Visium platform. In the original study, Xu *et al.* annotated the H&E image into 20 spatial domains that were grouped into four major histological types: ductal carcinoma in situ/lobular carcinoma in situ (DCIS/LCIS), invasive ductal carcinoma (IDC), tumor-edge regions with low malignancy, and normal tissue (Fig. 4A).

We compared SRLST with SCANPY, SpaGCN, STAGATE, DeepST, stLearn, SEDR, and PROST, and assessed domain identification accuracy using the adjusted rand index (ARI). SRLST achieved the highest ARI score (0.56), outperforming all other methods on the breast cancer dataset (Fig. 4B). Visualization of spatial clustering revealed that SRLST produced domain maps highly consistent with histological annotations (Fig. 4C). The boundaries between tumor regions were continuous and sharply defined, reflecting coherent spatial organization. Notably, SRLST was the only method that completely delineated the large IDC_5 region, whereas other approaches yielded fragmented or partially merged segmentations.

To further assess the biological relevance of the identified domains, we examined spatial expression patterns of four breast cancer related genes, CPB1, CRISP3, COX6C, and S100G, which were selected by differential expression analysis (Fig. 4D). These genes are well-established markers of subtype differentiation and prognosis: CPB1 is enriched in DCIS and distinguishes it from other subtypes (Bolkestein *et al.* 2020, Tokura *et al.* 2022); CRISP3 is linked to tumor progression and therapeutic resistance (Wang *et al.* 2022); COX6C shows elevated expression in breast cancer tissues and correlates with poor outcomes (Liu *et al.* 2024); and S100G has been reported as a prognostic indicator (Cancemi *et al.* 2018). The spatial domains identified by SRLST showed strong concordance with the expression patterns of key marker genes. Regions corresponding to DCIS/LCIS (domains 1, 4, 5, and 12) were enriched for CPB1, COX6C, and S100G, whereas IDC regions (domains 2 and 9) exhibited high CRISP3 expression (Fig. 4E). Notably, SRLST was the only method that completely recovered the COX6C-enriched domain 4.

Collectively, these findings show that SRLST provides more accurate spatial domain delineation and faithfully captures the molecular heterogeneity within the tumor microenvironment.

### 3.5 SRLST maps spatially organized tumor and non-tumor compartments in zebrafish melanoma

**Figure 5:**
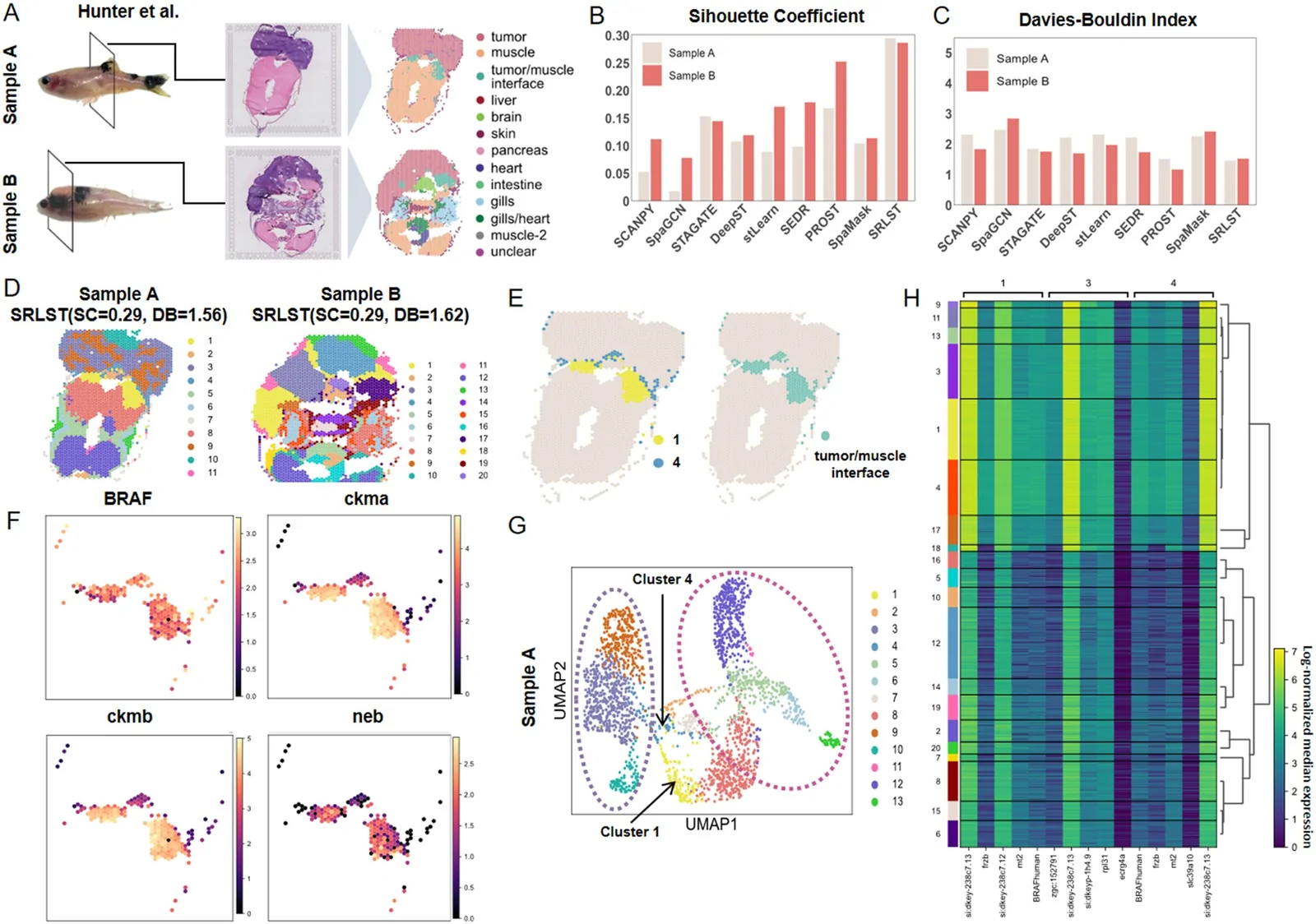
High-resolution spatial analysis of zebrafish melanoma tissue. (A) H&E images and expert annotations of zebrafish melanoma samples A and B. (B) SC score comparison of all methods on samples A and B. (C) DB score comparison of all methods on sample A and B. (D) Spatial clustering results of SRLST on samples A and B. (E) Location of tumor–muscle interface and corresponded clusters identified by SRLST on sample A. (F) Spatial expression patterns of BRAF, ckma, ckmb, and neb. (G) UMAP visualization of clustering results from SRLST on samples A and B. Tumor and non-tumor clusters are indicated by dotted outlines (left for tumor and right for non-tumor). (H) Heatmap of the top five differentially expressed genes in clusters 1, 3, and 4 for sample B.

The tumor microenvironment plays a critical role in driving tumor invasion and progression. To evaluate the ability of SRLST to resolve spatial interactions between tumor and surrounding normal tissues, we analyzed the zebrafish melanoma dataset reported by Hunter *et al.* (2021). The dataset includes two zebrafish samples (A and B), each capturing melanoma and adjacent non-tumor regions spanning multiple organs, including muscle, liver, and brain (Fig. 5A). We set the number of spatial clusters to 13 for sample A and 20 for sample B and assessed clustering performance using the Silhouette Coefficient (SC) (Shahapure and Nicholas 2020) and Davies–Bouldin Index (DB) (Vergani and Binaghi 2018), where a higher SC and a lower DB indicate better cluster separation.

In sample A, SRLST achieved the highest SC and the lowest DB scores, indicating the best overall clustering performance (Fig. 5B and C). PROST and STAGATE showed comparable but slightly inferior results. In contrast, SpaGCN yielded the lowest SC and the highest DB score, suggesting that its integration of spatial information might not have effectively captured the true tissue structure. A similar trend was observed in sample B, where SRLST and PROST again achieved the best performance, characterized by high SC and low DB values. SpaGCN consistently produced the poorest scores across both samples. Visualization also showed that the identified spatial domain of SRLST closely matched the histological annotations and accurately delineated major organs, including the brain, gills, and heart, within the complex tissue organization of sample B (Fig. 5D).

We next examined SRLST’s ability to distinguish tumor and adjacent non-tumor tissues. In sample A, the model identified two clusters (clusters 1 and 4) corresponding to the tumor–muscle interface (Fig. 5E). Differential expression analysis revealed distinct enrichment patterns: cluster 1, located near muscle tissue, showed high expression of muscle marker genes (ckma, ckmb) (Qiu *et al.* 2016, Jaffe *et al.* 2021), while cluster 4, adjacent to tumor tissue, expressed tumor-associated genes such as BRAF (Ribas and Flaherty 2011) and neb (Wang *et al.* 2020) (Fig. 5F). UMAP visualization confirmed that clusters 1 and 4 were spatially adjacent, with their embeddings forming a continuous transition from muscle to tumor regions (Fig. 5G).

In sample B, SRLST further subdivided the tumor compartment into three transcriptionally distinct subclusters (clusters 1, 3, and 4). Differential expression analysis revealed frzb and mt2 as marker genes for clusters 1 and 4. Prior studies report that overexpression of frzb can inhibit tumor cell proliferation, migration and invasion, and it has been proposed as a prognostic biomarker and potential therapeutic target (Liu *et al.* 2022). mt2 is a key regulator of melanocyte formation (Xia *et al.* 2022). In cluster 3, RPL31 was specifically upregulated, consistent with its previously reported role in tumorigenesis (Wu *et al.* 2023).

Taken together, these marker patterns point to intra-tumor heterogeneity: clusters 1 and 4 appear to engage melanocyte-associated programs with restrained proliferative potential, whereas cluster 3 exhibits a transcriptional signature consistent with increased tumorigenic activity.

### 3.6 SRLST resolves fine-grained spatial domains in mouse brain tissue under complex multi-region settings

**Figure 6:**
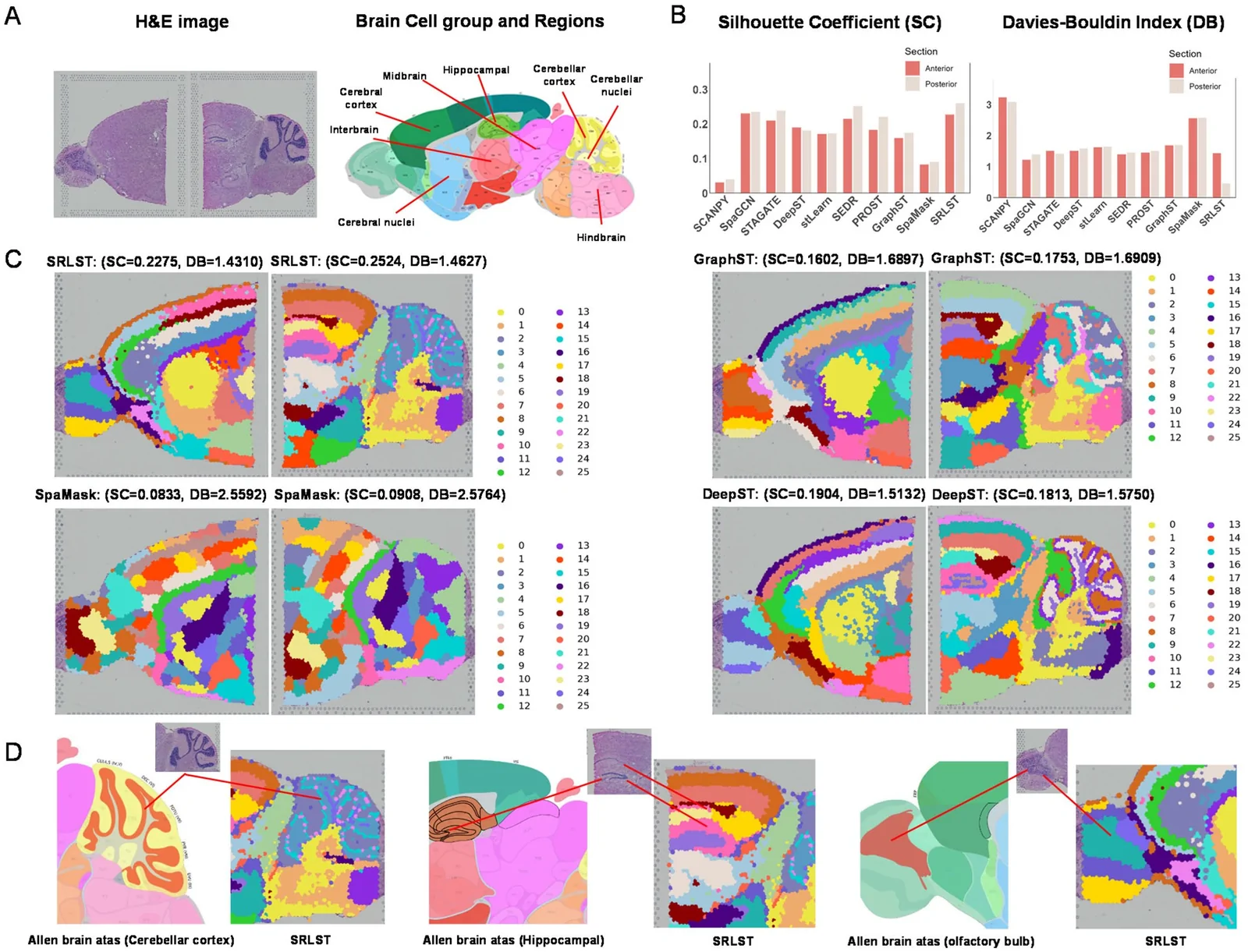
Comprehensive evaluation of SRLST on mouse brain two-slice joint and spatial domain identification. (A) H&E image of two adjacent mouse brain sections (anterior and posterior) and brain cell group and regions annotations from the Allen Mouse Brain Atlas (available from https://mouse.brain-map.org/static/atlas). (B) SC and DB score comparison of all methods on Section anterior and Section posterior. (C) Spatial clustering results of all SRLST, GraphST, SpaMask and DeepST. (D) Spatial domains of mouse brain tissue anterior and posterior regions.

To evaluate method performance under complex multi-region brain tissue, we applied SRLST to two adjacent mouse brain sections (anterior and posterior). Using the Allen Mouse Brain Atlas (Allen Institute for Brain Science 2008) annotation (Fig. 6A) as reference, we set the number of spatial clusters to 26 for both sections. First, we assessed clustering performance via SC and DB metrics.

In Section anterior, SRLST, SEDR, and SpaGCN achieved the best performance, with the highest SC and lowest DB scores among all compared methods (Fig. 6B). Across both Sections, Scanpy yielded the lowest SC and the highest DB score, suggesting that its integration of spatial information might not have effectively captured the true tissue structure. In Section posterior, SRLST achieved the highest SC and the lowest DB indicating the best overall clustering performance.

We next examined the ability of different methods to distinguish fine-grained subregions across the anterior and posterior sections. SRLST successfully identified fine-grained anatomical structures across both sections, including distinct brain Stem fields (Interbrain, Midbrain, and Hindbrain), cerebrum fields (Cerebral cortex and Cerebral nuclei), and cerebellum (Cerebellar cortex and Cerebellar nuclei). In contrast, Spamask failed to resolve these fine-grained regions, exhibiting fragmented or incomplete delineation (Fig. 6C). For the cerebellar cortex, both GraphST and DeepST successfully identified this region, but GraphST produced an incomplete delineation. For the hippocampal region, SRLST achieved the closest alignment with the H&E image among all compared methods. Move over, SRLST clearly detected the gyrus sections of the cerebellar cortex, the hippocampal region in the posterior and the olfactory bulb in the anterior (Fig. 6D), which is consistent with the reference annotations and H&E image.

Under these demanding conditions, SpaGCN produced severely fragmented clusters with poor alignment across the section boundary. STAGATE achieved smoother segmentation but failed to identify key subregions, including the dorsal and ventral horns of the hippocampus. GraphST showed moderate integration capability but exhibited incomplete boundary delineation in several subregions. In contrast, SRLST uniquely achieved both seamless multi-slice alignment and accurate resolution of fine-grained anatomical structures, preserving spatial continuity across section boundaries while capturing hippocampal subfields, cortical layers, and cerebellar cortex. These results demonstrate the robustness of SRLST in multi-slice integration and clustering tasks.

## 4 Discussion

Spatial transcriptomics enables the study of gene expression within its native anatomical organization context, but accurately capturing continuous and discontinuous spatial structures while preserving multi-modal heterogeneity remains challenging. To address this challenge, we propose SRLST, a unified representation learning framework that integrates spatial coordinates, transcriptomic profiles, and histology image derived tissue features through jointly trained graph embeddings. Across five biologically distinct datasets, SRLST consistently improved spatial domain resolution, boundary delineation, and biological interpretability, demonstrating strong generalization to complex tissue organizations.

SRLST achieves these improvements by jointly modeling spatial proximity, gene expression similarity, and morphological features extracted from histology images. In contrast to approaches that rely solely on molecular profiles or assume smooth spatial continuity, SRLST incorporates tissue boundary information into graph construction, enabling accurate detection of small or fragmented domains. The fusion of complementary embeddings allows the model to maintain continuous anatomical structures while preserving biologically meaningful discontinuities, thereby generating representations that more faithfully reflect tissue organization.

Although SRLST improves spatial representation across diverse tissues, it is currently optimized for spot-level data and may require adaptation for single-cell–resolution platforms. Its reliance on H&E-derived features also makes performance sensitive to staining variation. Future work will focus on enhancing robustness, improving scalability to larger tissues, and extending the framework to incorporate additional spatial omics modalities to better characterize cellular interactions and disease progression. In particular, recent advances in single-cell data analysis provide valuable insights for these extensions. For instance, scMCG (Lan *et al.* 2025b) leverages contrastive learning and generative adversarial networks to effectively capture cellular heterogeneity from chromatin accessibility data, while deep imputation bi-stochastic graph regularized matrix factorization (Lan *et al.* 2026b) addresses the challenge of missing values in scRNA-seq clustering through a graph-regularized framework. These strategies offer promising directions for adapting SRLST to emerging spatial epigenomic and sparse datasets.

## Data Availability

All processed datasets analyzed in this study (DLPFC, fish, human liver cell and human breast cancer) have been deposited into zenodo (https://zenodo.org/records/15624210).

## Code availability

The SRLST algorithm is implemented in Python and is publicly available at https://github.com/lanbiolab/SRLST.
